# Social stimulation and corticolimbic reactivity in premenstrual dysphoric disorder: a preliminary study

**DOI:** 10.1186/2045-5380-4-3

**Published:** 2014-02-26

**Authors:** Malin Gingnell, Victoria Ahlstedt, Elin Bannbers, Johan Wikström, Inger Sundström-Poromaa, Mats Fredrikson

**Affiliations:** 1 Department of Women’s and Children’s Health, Uppsala University, Uppsala, Sweden; 2 Department of Psychology, Uppsala University, Uppsala, Sweden; 3 Department of Radiology, Oncology and Radiation Science, Uppsala University, Uppsala, Sweden

## Abstract

**Background:**

Premenstrual dysphoric disorder (PMDD), characterized by luteal phase-induced negative affect and loss of impulse control, often results in compromised social interactions. Although amygdala activation is generally linked to negative affect, increased amygdala reactivity to aversive stimuli in the luteal phase has not been consistently reported in PMDD. We tested the hypothesis that amygdala hyper-reactivity in PMDD is symptom specific, rather than generalized, and linked to socially relevant stimuli. Blood oxygenation level dependent signal changes during exposure to negative images with social and non-social content were evaluated in the mid-follicular and late luteal phase of the menstrual cycle. Fourteen women with PMDD and 13 healthy controls participated.

**Results:**

When compared with healthy controls, women with PMDD in the luteal phase had enhanced reactivity to social stimuli compared to non-social stimuli in the amygdala and insula, but attenuated reactivity in the anterior cingulate cortex. Functional couplings between emotion processing and controlling areas were significantly different, being positive in women with PMDD and negative in healthy controls. Changes in progesterone levels in women with PMDD correlated positively with altered amygdala reactivity.

**Conclusions:**

Socially relevant aversive stimulation elicited enhanced activity in affective processing brain regions that were functionally coupled to compromised activity in cognitive control areas. Because increased reactivity correlated positively with alterations in ovarian steroid levels, data preliminary support the hypothesis that enhanced progesterone sensitivity in PMDD affects corticolimbic processing of social emotions.

## Background

Premenstrual dysphoric disorder (PMDD), characterized by luteal phase-induced anxious and depressive symptoms as well as emotional lability [1], affects around 5% of women of reproductive age [2]. The symptomatology compromises quality of life, including social interactions [3]. Because the core symptoms of PMDD are affective in nature, it has been suggested that brain areas in the fear circuit [4], particularly the amygdala, mediate PMDD symptoms [5]. The amygdala, insula and anterior cingulate cortex (ACC) form a hypothesized corticolimbic emotion processing network [4,6], with the amygdala and insula activated by bottom-up emotional processes, and the ACC involved in top-down regulation [4,7]. Although increased amygdala reactivity characterizes negative affective states like anxiety and depression [8-15], studies on amygdala reactivity in PMDD are inconsistent [16-18]. Protopopescu *et al*. [16] reported increased amygdala reactivity in response to emotional words, but their results reflected alterations in reactivity over the menstrual cycle in healthy controls rather than in women with PMDD. Gingnell *et al.*[17] also reported a luteal phase-induced increase in amygdala reactivity to emotional faces, but only among a subgroup of patients with PMDD with high trait anxiety [17]. Furthermore, Gingnell *et al*. [18] observed increased reactivity to negative emotional stimuli in the amygdala and insula, but no differences between patients and controls and with no menstrual phase modulation.

Some anxiety disorders are characterized by a generally altered emotional reactivity. In post-traumatic stress disorder (PTSD), for example, increased amygdala reactivity and decreased activity in emotion controlling areas is observed not only in response to trauma-related challenge but also to non-symptomatic stimulation such as aversive emotional faces, even outside awareness [19-21]. By contrast, in other disorders like specific phobia, amygdala hyper-reactivity is circumscribed to phobic cues and absent in response to other emotionally relevant situations [22]. It has not been determined if amygdala reactivity in women with PMDD reflects a generally altered emotional responsivity or whether exaggerated amygdala reactivity is specific to symptomatic challenges. PMDD symptoms compromise everyday social functions both at work and at home, resulting in frequent reports of disrupted interpersonal interactions [23]. Although it is uncertain if partner violence is a significant risk factor for PMDD [24-26], both women with a history of trauma and with PTSD are more likely to experience PMDD, especially when trauma exposure involves interpersonal violence [27,28]. In addition, women with PMDD with a history of trauma have abnormal neuroendocrine stress responses compared to women with PMDD without a trauma history [24-26,29].

Because PMDD symptoms affect social interactions and perceived social threat might be crucial for the development of the disorder, it is conceivable that enhanced amygdala reactivity is elicited mainly by socially relevant stimuli. Emotional words and general emotional stimuli [16,17] might not tap into PMDD symptomatology, and therefore not increase amygdala reactivity. If so, mixing generally emotion-arousing stimulation with more focused symptomatic challenges may produce inconsistent results, such as those previously reported [16-18]. Furthermore, even though the amygdala has a key role in anxiety and negative affect, both theoretical reasoning [30] and empirical results [8,15] support that other areas in the fear circuit [4], such as the insula, and cognitive control areas, like the ACC, are involved in emotional processing.

We hypothesized that socially relevant stimulation is a prime elicitor of negative affect in PMDD, reflected in corticolimbic circuit functions with increased brain reactivity in the affective processing regions of the amygdala and insula [4,6-8,15,30], as well as decreased reactivity in the regulatory ACC region [31] and an altered functional coupling between the processing and regulatory areas [31]. Because PMDD symptoms include negative emotional symptoms that are experienced in the luteal but not the follicular phase of the menstrual cycle, altered reactivity and connectivity should be evident predominantly in the luteal phase and possibly coupled to variations in ovarian steroid hormones [5,32]. This hypothesis was tested by evaluating brain reactivity and connectivity using functional magnetic resonance imaging (fMRI) of blood oxygenation level-dependent (BOLD) activity to social and non-social negative stimuli in the follicular and luteal phase of the menstrual cycle in women with PMDD and healthy controls. We also explored if ovarian steroid hormones correlated with corticolimbic circuit functions.

## Methods

We re-analyzed data from a study including social and non-social aversive emotional pictures [18]. The original paradigm included exposure to emotional images of negative or positive valence. All emotional-image slides were proceeded either by a red cue, signaling negative affect, or a green, associated with positive pictures. The timing was such that the color cue was displayed 5 s before a 2 s exposure of the social slide, and followed by a 2.5 to 3.5 s black screen with a inter-trial interval of 9 to 11 seconds.

The emotional stimuli, 15 negative and 15 positive pictures, were selected from the International Affective Pictures System (IAPS) [33]. For an example of the paradigm see Additional file 1. We analyzed BOLD responses to socially relevant and irrelevant negative emotional stimuli. Negative stimuli were chosen because PMDD mainly comprises negative emotional symptoms [1].

### Participants

Seventeen women with PMDD and 16 asymptomatic controls were recruited through a newspaper advertisement and from women with a PMDD diagnosis.

PMDD was diagnosed according to the definitions in the Diagnostic and Statistical Manual of Mental Disorders IV [1]. Details of the diagnostic procedure have been described previously [34]. Briefly, prospective ratings of daily symptoms using the Cyclicity Diagnoser (CD-scale) were completed to confirm the presence of PMDD and to estimate the severity of PMDD symptoms. The number of days during the 10 days before menses when participants reported a score of 2 or more on the CD-scale for each of the the four core symptoms of PMDD (irritability, depression, anxiety and mood swings) (i.e. a scale 0–40) [35], and number of days when social interaction was avoided (0 to 10) were used as measures of PMDD severity. The asymptomatic controls were physically healthy women with regular menstrual cycles and no history of premenstrual dysphoric symptoms. None of the controls reported premenstrual dysphoric symptoms on daily ratings. The study was approved by the Ethical Review Board of Uppsala, Sweden, and all participants gave written informed consent.

Exclusion criteria were pregnancy; treatment with hormonal compounds or psychotropic drugs; or presence of any ongoing psychiatric disorder. Absence of other psychiatric disorders was confirmed using the structured psychiatric interview, Mini International Neuropsychiatry Interview [36]. Furthermore, participants with pacemakers, cardiac defibrillators, aneurysm clips, cochlear implants or other implants including magnets, batteries or wires were excluded. One woman with PMDD and one healthy control dropped out after the first scanning session due to personal reasons, and two healthy controls and three women with PMDD were excluded due to movement artifacts (peaks of movement in the x/y/z-axis of more than 3 mm or more than 2 degrees of rotation), or incomplete scanning sessions due to hardware problems. There were no significant differences in demographic or behavioral data between excluded and remaining participants. Fourteen women with PMDD and 13 healthy controls were analyzed.

### Timing according to the menstrual cycle

fMRI scanning was performed twice, once in the mid-follicular phase (6 to 12 days after the onset of menstrual bleeding) and once to coincide with the late luteal phase (postovulatory day 8 to 13), according to a positive luteinizing hormone assay (Clearplan, Unipath, Bedford, UK). Monitoring of the luteal phase was confirmed by progesterone serum concentrations and records of the next menstrual bleeding. The study was counterbalanced across the menstrual cycle with half of the participants scanned first in the follicular phase and then in the luteal phase, and the other half scanned in the reverse order.

### Hormonal analyses

Blood samples were drawn before each scanning. Estradiol and progesterone serum concentrations were determined by competitive immunometric electrochemistry luminescence detection at the Department of Clinical Chemistry, Uppsala University Hospital. The samples were run on a Roche Cobas e601 with Cobas Elecsys reagent kits (Roche Diagnostics, Bromma, Sweden). The measurement interval was 0.1 to 191 nmol/l for progesterone and 18.4 to 15,781 pmol/l for estradiol. The progesterone intra-assay coefficient of variation was 2.21% at 2.39 nmol/l and 2.82% at 31.56 nmol/l. The estradiol intra-assay coefficient of variation was 6.8% at 85.5 pmol/l and 2.8% at 1,640 pmol/l.

### Mood and anxiety scales

Prior to each fMRI scan, participants completed the self-rated version of the Montgomery-Åsberg Depression Rating Scale (MADRS-S) [37] and the state portion of the Spielberger State-Trait Anxiety Inventory (STAI-S) [38].

### Functional magnetic resonance imaging - scans and paradigm

fMRI was performed using a 3 T whole body scanner (Achieva 3 T X Philips scanner Philips Medical Systems, Best, The Netherlands) equipped with an eight-channel head coil. At the beginning of each scanning session, an anatomical T_1_-weighted reference data set to a voxel size of 0.8 × 1.0 × 2.0 mm^3^ and 60 slices were acquired. During stimulus presentations, BOLD imaging was performed using a single shot echo-planar imaging sequence with parameters echo time/repetition time 35/3,000 ms, flip angle 90°, acquisition matrix 76 × 77, acquired voxel size 3.0 × 3.0 × 3.0 mm^3^ and 30 slices.

The participants lay facing upwards in the scanner with their heads lightly fixated. Visual stimuli were presented through goggles mounted on the head coil (VisualSystem, NordicNeuroLab, Bergen, Norway). The stimulus paradigm was implemented using the commercial software package E-prime (Psychology Software Tools, Sharpsburg, PA, USA). To synchronize the paradigm and the MR sequence, a SyncBox (NordicNeuroLab) was used. The paradigm included 15 negative pictures selected from the IAPS [33] preceded by a color cue indicating the valence. We compared the eight slides displaying negative social situations (for example, injured humans, abduction of a young female; IAPS: 3320, 2710, 3051, 3160, 6312, 6570, 8230, 9042) with the seven pictures containing negative, but non-social stimuli (for example, snakes, threatening dogs; IAPS: 1050, 1052, 1111, 1201, 1274, 1525, 9620). After scanning, participants again viewed and rated pictures for valence and arousal using the Self-Assessment Manikin used in the IAPS material [33]. Arousal ratings are available in Additional file 2 but are not included here, as we did not test any arousal-related hypotheses. The valence ratings for social and non-social stimuli were analyzed in a Group by Phase analysis of variance, with additional follow-up *t*-tests.

### Functional magnetic resonance imaging - preprocessing and analysis

The Digital Imaging and Communications in Medicine images from the scanner were converted to Neuroimaging Informatics Technology Initiative files using the freeware package MRicron [39]. The data were then analyzed in MatLab (MathWorks, Natick, MA, USA) using SPM5 [40]. The individual BOLD images were realigned to a mean image for the session, slice timed to the middle slice of each whole brain volume, co-registered with the individual anatomic scan, normalized into Montreal Neurological Institute (MNI) coordinates space using normalization parameters obtained from a segmentation into the white matter, grey matter and cerebrospinal fluid of the individual anatomical scan, and smoothing was performed using an 8 mm kernel.

For each individual, BOLD signal changes in the fMRI time series were regressed on to social and non-social negative images. Onsets and durations for stimuli included in the paradigm but not analyzed in the present study (that is, anticipatory periods, positive emotional stimuli) and the six movement parameters obtained in the realignment step were included in the model. Contrast maps were calculated for each individual of the contrast between social and non-social negative images. These contrast maps were then used for group comparisons. Analyses of group differences were first performed to compare women with PMDD and healthy controls during the luteal phase. Regions of interest (ROIs) were generated using the automatic anatomical labeling definitions in the Wake Forest University School of Medicine PickAtlas [41-43] and included the bilateral amygdala, insula and ACC. Then, a ROI defined by the group differences observed in the luteal phase was used for between-group comparisons in the follicular phase and for within-group comparisons between phases. To test the *a priori* hypothesis of increased reactivity in the amygdala and insula as well as attenuated reactivity in the ACC in PMDD during the luteal phase, an uncorrected *p*-value of 0.05 with *k* ≥5, corrected for the search volume of each ROI, was used. Functional couplings during the luteal phase between the amygdala and the insula, respectively, to the ACC, were evaluated with extracted data from the significant clusters, as defined by the between-participants effects in the luteal phase, used as seeds for correlations. These analyses were performed in each group separately. The relatively lenient statistical threshold was deliberately chosen as we restricted analyses only to ROIs where specific hypotheses were advanced. This approach does not only focus on type I errors but also gives a balance between type I and type II errors [44,45].

Self-reports and affective picture ratings were compared by paired and independent *t*-tests, respectively. Estradiol and progesterone levels were compared using Mann-Whitney *U* test and Wilcoxon signed rank tests, respectively. Symptom severity and number of days when social interaction was avoided were evaluated using Student’s *t*-tests. In addition, partial correlations adjusted for affective ratings were performed between alterations in brain reactivity and change in ovarian steroid hormone levels (follicular to luteal phase) to evaluate if brain activity was tied primarily to changes in hormonal activity or subjective ratings.

## Results

### Demographics and hormonal results

**Figure 1:**
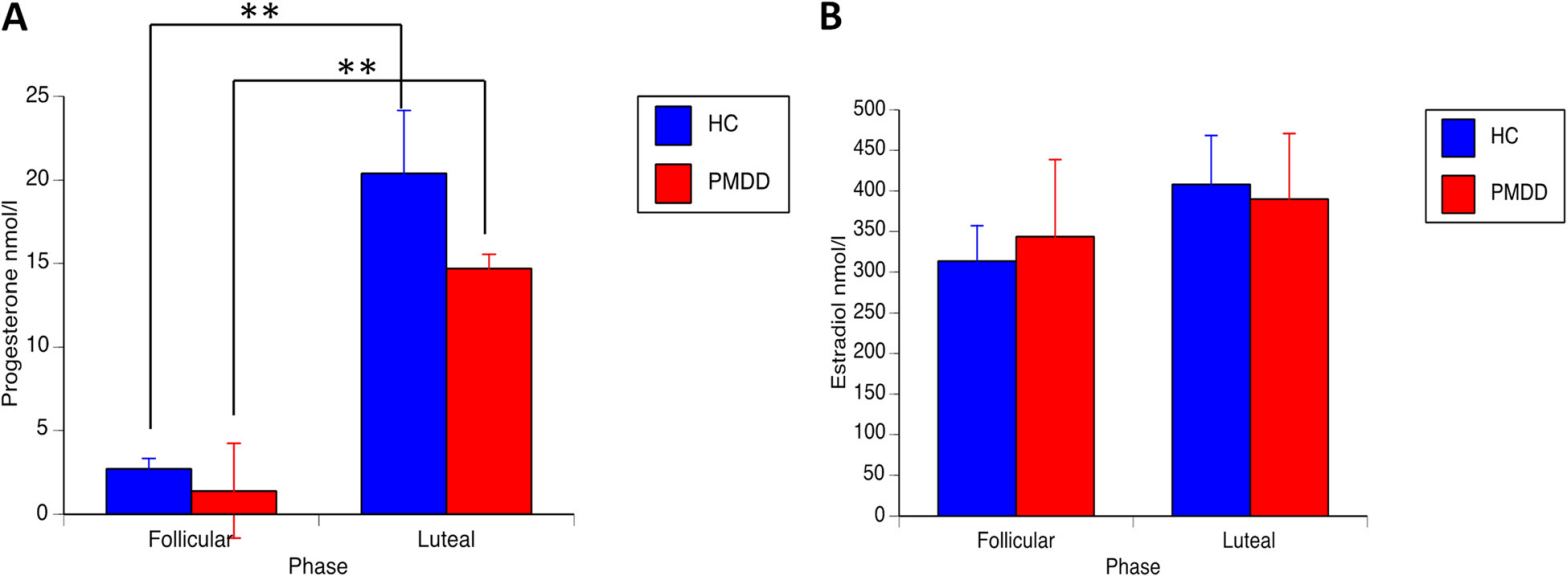
**Hormone levels. (A)** Progesterone and **(B)** estradiol levels in women with PMDD and healthy controls during the follicular and luteal phase of the menstrual cycle. In both groups, progesterone increased significantly in the luteal phase whereas estradiol was similar across phases. No significant group differences emerged in either phase. **p <0.001. HC, healthy controls; PMDD, premenstrual dysphoric disorder.

No significant group differences emerged for age (PMDD 35.0 ± 8.9 years; healthy controls 33.1 ± 7.8 years; *t*(25) = 0.6; *p* = 0.56), day of testing in the follicular phase (PMDD 8.5 ± 1.9; healthy controls 10.1 ± 3.5; *t*(25) = 1.8; *p* = 0.084), or luteal phase (PMDD −4.6 ± 3.8, healthy controls −4.4 ± 2.7; *t*(25) = 0.35; *p* = 0.73). Similarly, hormonal levels did not differ between groups for follicular phase progesterone (*U* = 52.5, *p* = 0.062), luteal phase progesterone (*U* = 68.0, *p* = 0.28), follicular phase estradiol (*U* = 75.0, z = −0.77, *p* = 0.44) and luteal phase estradiol (*U* = 77.5, z = −0.66, *p* = 0.51). Estradiol levels were similar in the follicular and luteal phase in both groups (for both groups *Z* <0.87, *p* >0.38). However, progesterone increased significantly from the follicular to the luteal phase in both groups (healthy controls *Z* = 2.9, *p* = 0.004; and PMDD *Z* = 3.3; *p* = 0.001; Figure 1).

### Behavioral results

**Figure 2:**
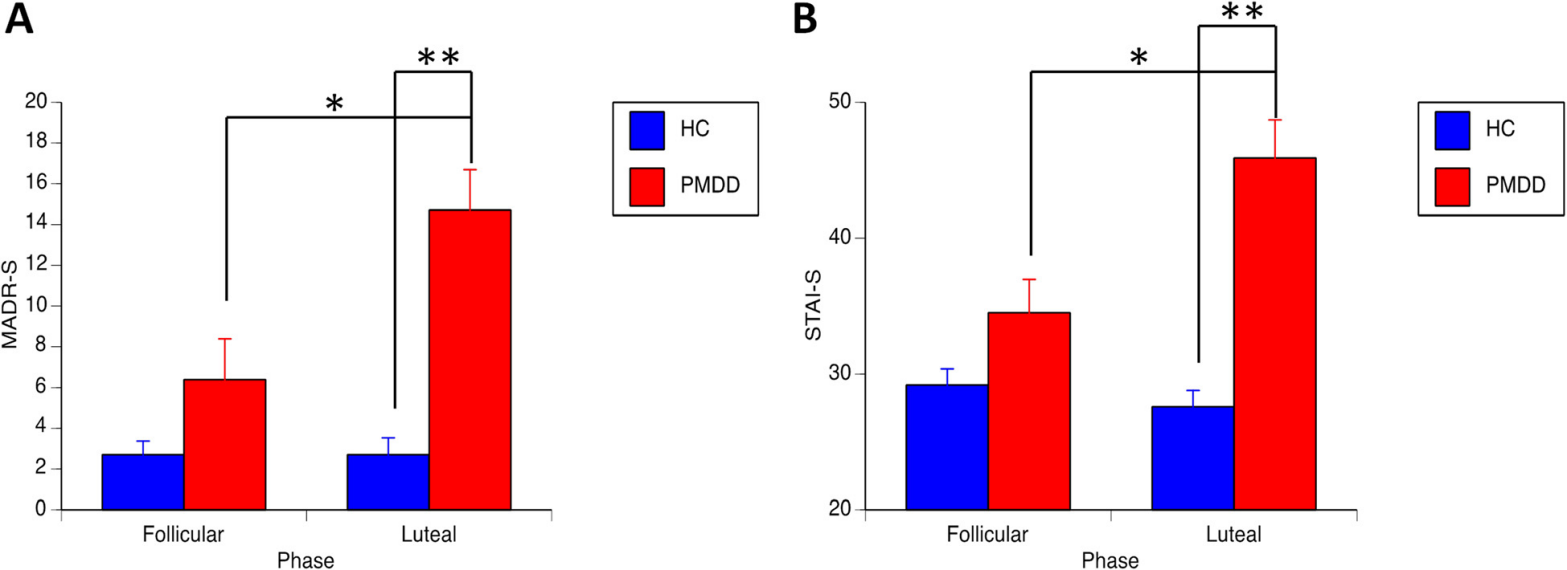
**Depression and anxiety ratings. (A)** MADRS-S and **(B)** STAI-S self-report ratings for women with PMDD and healthy controls during the follicular and luteal phase of the menstrual cycle. Women with PMDD had higher scores than healthy controls during the luteal phase and higher in the luteal than the follicular phase. No significant group differences were present in the follicular phase or for healthy controls between phases. *p <0.05; **p <0.001. MADRS-S, Montgomery-Åsberg Depression Rating Scale - self-rated version; STAI-S, State-Trait Anxiety Inventory - self-rated version PMDD, premenstrual dysphoric disorder; HC, healthy controls.

Women with PMDD had higher MADRS-S and STAI-S scores during the luteal compared to the follicular phase (*t*(13) = 2.7, *p* = 0.017 and *t*(13) = 2.5, *p* = 0.027, respectively) whereas in healthy controls luteal phase ratings did not differ from the follicular phase (for both measures *t*(13) <1.1, *p* >0.27). When compared to healthy controls, women with PMDD scored higher on MADRS-S (*t*(25) =5.4, *p* <0.0001) and STAI-S (*t*(25) =5.7, *p* <0.0001) in the luteal phase but not in the follicular phase (for both measures *t*(25) <1.8, *p* >0.078; Figure 2). Women with PMDD had a symptom severity of 27.9 ± 2.3 (range 0 to 40) [35] and avoided social interaction during 5.1 ± 1.0 out of 10 premenstrual days. Corresponding values for healthy controls were 8.1 ± 2.5 and 1.3 ± 0.6, respectively. The group differences were statistically significant for both measures (symptom severity: *t*(25) =5.6, *p* <0.0001; avoiding social interaction: *t*(25) = 3.2, *p* = 0.003.

**Figure 3:**
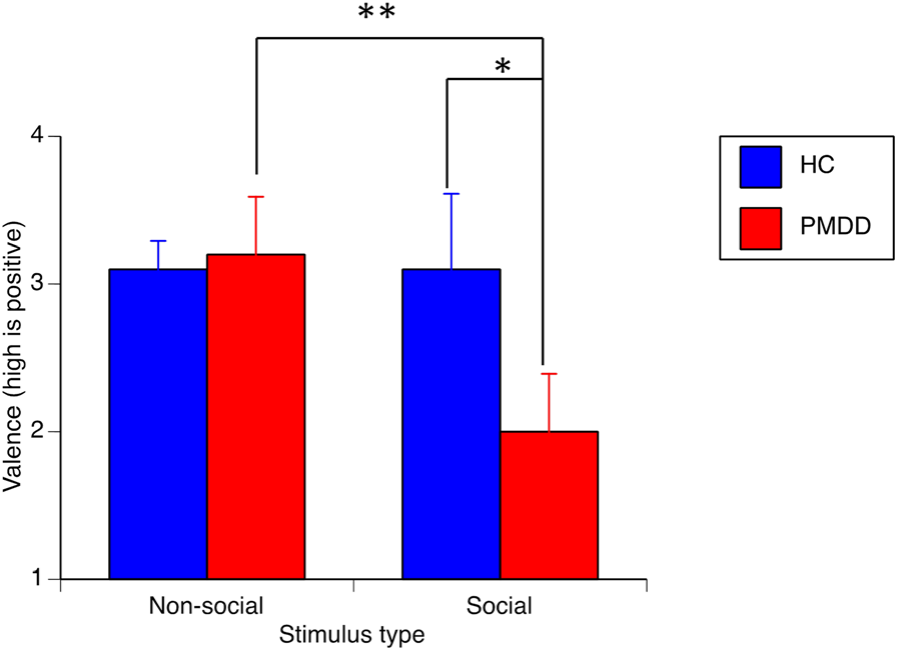
**Valence ratings in the luteal phase.** Women with PMDD rated images with social content as more negatively valenced than images with a non-social content, and rated social stimuli as more negative than did healthy controls. There were no group differences for ratings of non-social stimuli. *p <0.05, **p <0.001. HC, healthy controls; PMDD, premenstrual dysphoric disorder.

For valence ratings, the only significant difference was found for ratings of social stimuli in the luteal phase (*F* = 6.62, *p* = 0.017). Women with PMDD rated the social images significantly more negative than healthy controls during the luteal phase (*t*(24) =2.5, *p* = 0.021; Figure 3) but not in the follicular phase (*t*(25) =1.2, *p* = 0.24). Also, women with PMDD rated the social stimuli as more negative than the non-social stimuli both in the follicular (*t*(13) =3.4, *p* = 0.005) and the luteal phase (*t*(13) =4.3, *p* = 0.001; Figure 3), whereas healthy controls gave similar ratings for social and non-social stimuli during both phases (both phases *t* (13) <1.6, *p* >0.14). Arousal ratings are available in the Additional file 2: Table S1.

### Brain results

#### Between group comparisons of reactivity

**Figure 4:**
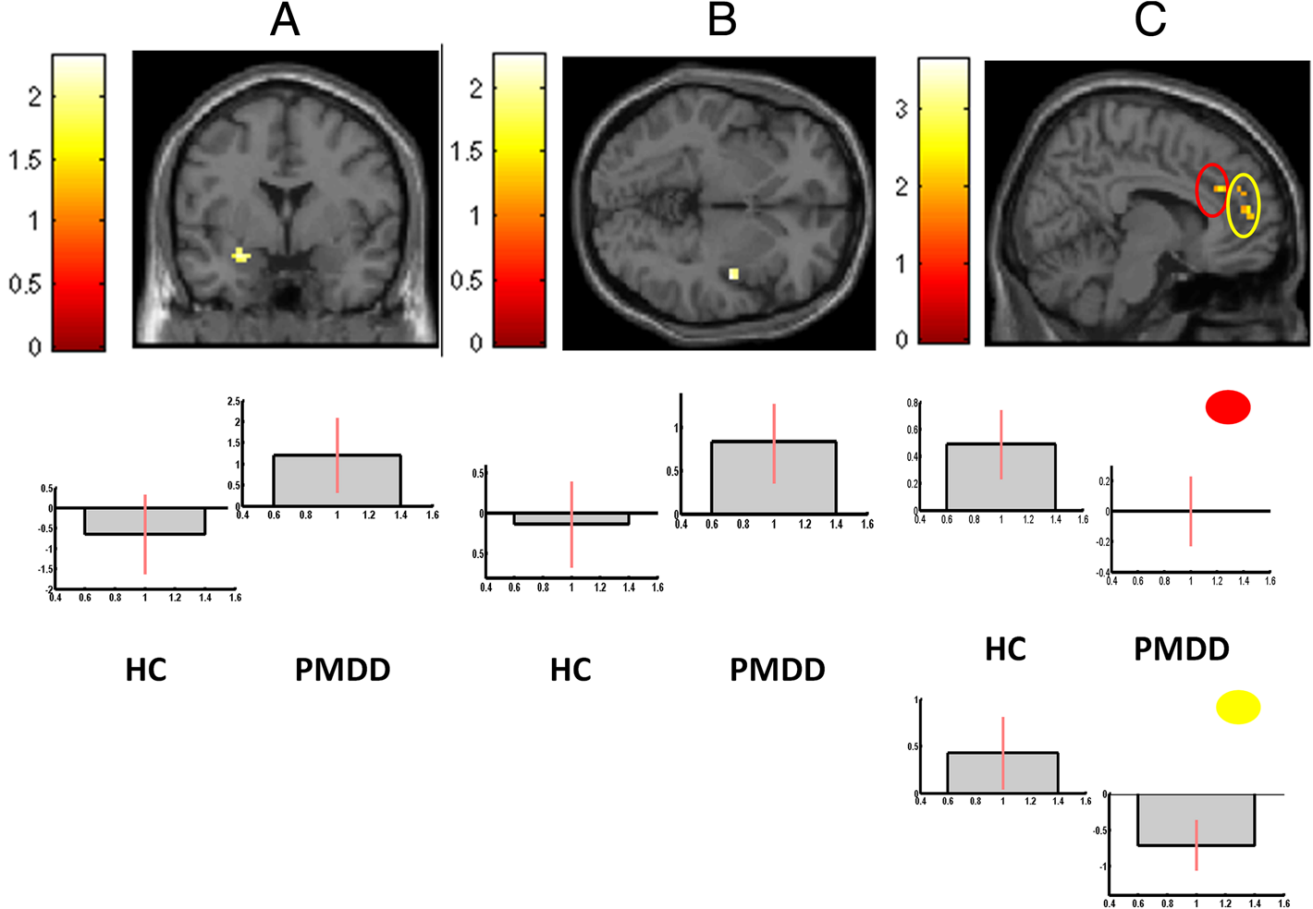
**BOLD reactivity.** Patients with PMDD had enhanced reactivity to socially relevant stimuli as compared to non-social stimuli in two regions of interest, the **(A)** left amygdala (−21, 2, −15; k = 11; z = 2.18; *P* = 0.015) and **(B)** the right insula (45, −9, −2, k = 10, z = 2.13, p = 0.016) when compared to healthy controls in the luteal phase. **(C)** Women with PMDD also had attenuated reactivity to social stimuli in the midline ACC in two clusters marked in red and yellow respectively (9, 33, 23; k = 12; z = 2.22; *p* = 0.013; and 3, 50, 11; k = 27; z = 3.23; *p* = 0.001). All anatomic localizations are given in Talairach coordinates. Brighter colors represent higher *t* scores. Below the brain images, contrast estimate plots are given for the peak voxel of each cluster. Healthy controls are given in the left panels and PMDD to the right. ACC, anterior cingulate cortex; PMDD, premenstrual dysphoric disorder.

During the luteal phase, women with PMDD had higher reactivity to social stimuli than healthy controls in the amygdala (−21, 2, −15; k = 11; z = 2.18; *p* = 0.015) and insula (45, −9, −2; k = 10; z = 2.13; *p* = 0.016), but lower reactivity in the ACC (two clusters: 9, 33, 23; k = 12; z = 2.22; *p* = 0.013; and 3, 50, 11; k = 27; z = 3.23; *p* = 0.001) (Figure 4). No group differences were observed in the follicular phase. The contrast between non-social and social images revealed no group differences in either phase.

#### Within group comparisons of reactivity

In women with PMDD, There was higher amygdala reactivity to social than non-social stimuli in the luteal as compared to the follicular phase (−21, 2, −15; k = 5; z = 1.94; *p* = 0.015). No phase differences were observed in healthy controls.

#### Connectivity

**Figure 5:**
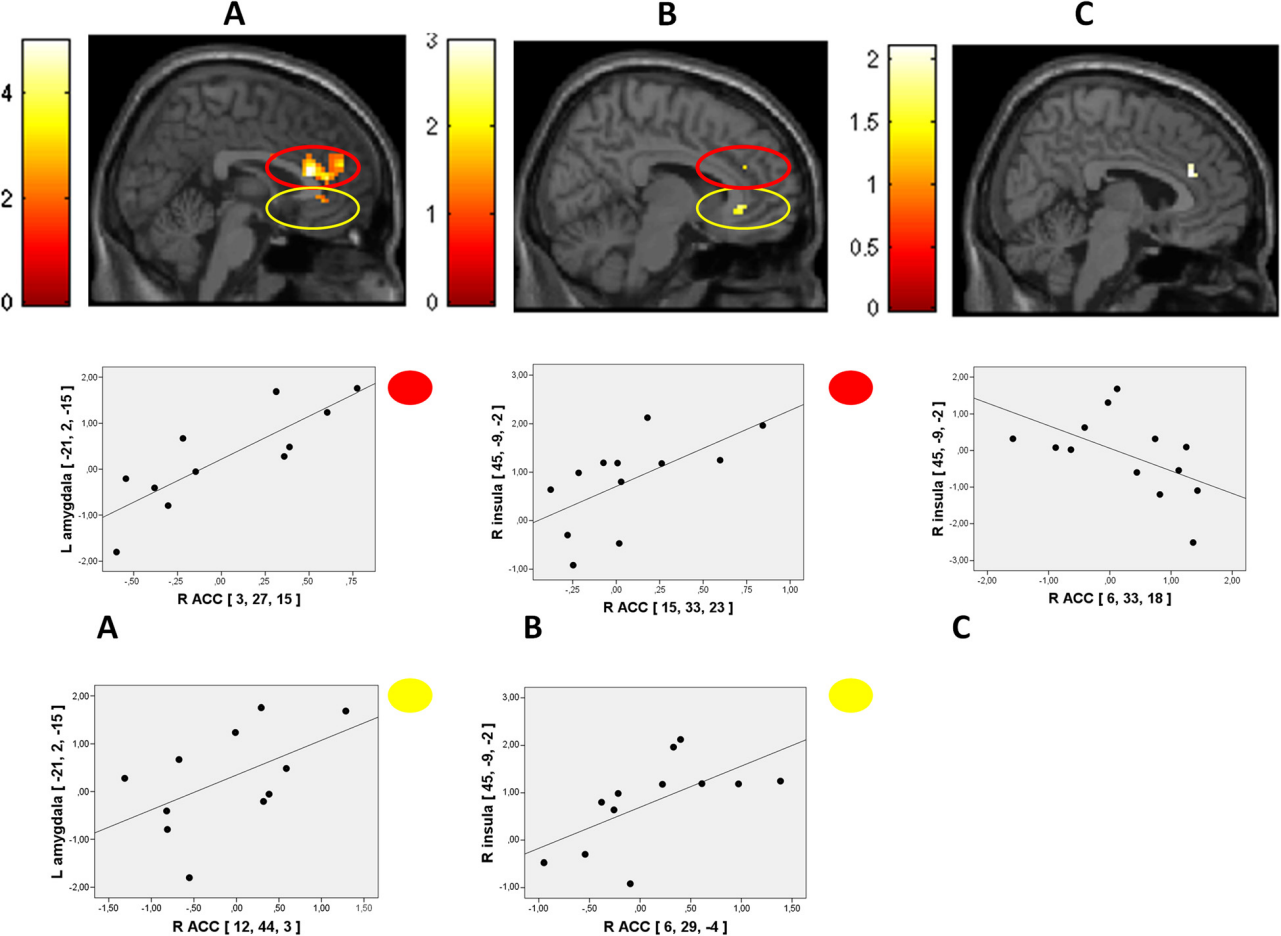
**Connectivity.** Connectivity analyses revealed a pattern of positive connectivity between BOLD reactivity in emotion processing and controlling areas in PMDD between the **(A)** amygdala and ACC (two clusters marked in red and yellow respectively: 3, 27, 15; k = 90; z = 3.53; *p* <0.001; and 12, 44, 3; k = 29; z = 2.68; *p* <0.016) and the **(B)** insula and ACC (two clusters marked in red and yellow respectively: 15, 33, 23; k = 7; z = 2.45; *p* = 0.008; and 6, 29, −4; k = 11; z = 2.46; *p* = 0.007). **(C)** For healthy controls there was a trend towards a negative relation between the ACC and insula (6, 33, 18; k = 9; z = 1.89; *p* = 0.029). All anatomic localizations are given in Talairach coordinates. Brighter colors represent higher *t* scores. Below the brain images, schematic representations of the connectivity are given for each cluster. Three outliers with BOLD reactivity >2 SD from mean of group were excluded from analyses. ACC, anterior cingulate cortex; PMDD, premenstrual dysphoric disorder.

Connectivity analyses revealed a pattern of positive connectivity between BOLD reactivity in emotion processing and controlling areas in PMDD (amygdala and ACC: 6, 45, 23; k = 5; z = 2.39; *p* = 0.008; and insula and ACC: 9, 33, 23; k = 6; z = 2.62; *p* = 0.004) whereas there was a trend towards a negative relation between the ACC and insula in healthy controls (6, 33, 23; k = 1; z = 1.74; *p* = 0.041). The pattern was identical when three outliers in BOLD reactivity (>2 SD from mean of group) were removed (Figure 5). The correlation strength between the insula and the ACC differed significantly between PMDD and healthy controls (z = 2.99; *p* = 0.0027).

#### Correlations with brain reactivity, ovarian steroids and affective ratings

For women with PMDD, the phase-related increase in amygdala reactivity to social as compared to non-social stimuli correlated positively with the corresponding change in progesterone level (r_xy_ = 0.61, *P* = 0.020). When partializing out the valence ratings, the correlation between progesterone and amygdala remained (r_xy_ = 0.63, *P* = 0.020).

## Discussion

We tested the hypothesis that women with PMDD are overly sensitive to negative social stimulation rather than generally affected by negative emotional stimuli and that this would be reflected in altered corticolimbic processing. Subjective reports confirmed an anxious and depressive state of mind, and sensitivity to social stimulation, in women with PMDD during the luteal phase. The negative feeling state was coupled to exaggerated reactivity in the amygdala and insula and attenuated reactivity in ACC regions projecting to the amygdala [46]. Amygdala reactivity was also higher in the luteal than the follicular phase. Collectively, data preliminary support the hypothesis that increased sensitivity to social stimulation characterizes PMDD and that corticolimbic circuit activity is altered more by socially relevant than irrelevant stimuli. Thus, previous inconsistent results on amygdala reactivity in PMDD [16-18] may reflect the use of a mixture of social and non-social stimuli.

The connectivity pattern must be regarded as preliminary due to the small sample size. Previous studies have reported functional couplings between the amygdala and the ACC [46] as well as between the ACC and insula [47,48]. The theoretically predicted negative functional couplings with enhanced reactivity in emotion processing areas associated with reduced reactivity in emotion regulating areas [31], consistent with top-down emotional control, was observed in healthy controls. By contrast, and in line with the hypothesis, women with PMDD displayed an aberrant connectivity pattern with positive couplings between both amygdala and insula reactivity on the one hand and ACC on the other, indicating the primacy of bottom-up processes. In social anxiety disorder, Klumpp and coworkers [49] recently reported that increased insula activation occurred simultaneously as ACC activity decreased, supporting an intrinsic relationship between the insula and the ACC. Conceptually similar results were reported for patients with social anxiety disorder, with decreased connectivity between the amygdala and rostral parts of the ACC to disorder-relevant stimuli [50], while studies in major depression report both reduced and enhanced connectivity between the ACC and amygdala [51]. The ACC areas with attenuated reactivity and compromised connectivity that was observed in our study are associated both with voluntary efforts to suppress emotional reactions [5] and with more automated regulatory processes [52,53]. Based on the present results, we cannot determine if voluntary or automated processes are implicated. Collectively, however, data support the bottom-up initiation of emotional reactions, rather than top-down control, in response to negative social stimulation in PMDD.

We did not seek to define the mechanisms through which luteal phase-determined corticolimbic processes to social stimuli in PMDD are altered. However, it could be that the subjective experience of social as compared to non-social stimuli in PMDD *per se* is of greater relevance to the patients and thus determines the increased amygdala reactivity. In support of this, we observed significant differences in subjective distress elicited by social but not non-social stimuli in the luteal phase between women with PMDD and healthy controls. However, patients with PMDD consistently rated social stimuli as more negative than non-social stimuli across both cycle phases, making it unlikely that alteration in experience is the sole mechanism driving the change in corticolimbic processing. In addition, amygdala reactivity over the course of the menstrual cycle did not correlate with alterations in affective ratings, but with progesterone levels. It is possible that amygdala reactivity in PMDD is a more sensitive measure than subjective ratings. This is in parallel to increased amygdala reactivity to emotional stimuli, without any relation to subjective reports, previously observed for carriers of the short version of the serotonin transporter promoter length polymorphism [54,55]. Our study may implicate that an overly sensitive threat detection system directed towards social stimuli could be a prerequisite for negative social interactions in PMDD during the luteal phase.

Another potential mechanism that may influence amygdala sensitivity over the menstrual cycle is alterations in progesterone levels [5,32]. Progesterone increased to a similar extent between the follicular and luteal phase both in women with PMDD and in healthy controls, but the increase in amygdala reactivity and the corresponding change in progesterone levels were positively correlated only in women with PMDD. Analyses disentangling phase-determined alterations in affective ratings from progesterone changes further supported the notion that hormonal alterations and not subjective experiences were coupled to amygdala reactivity. This indicates that individual differences in central nervous system activity over the menstrual phase are linked to ovarian steroid hormones rather than subjective experiences. As progesterone levels did not increase more in PMDD than healthy controls and since no change in ACC reactivity was observed across phases, data support the theory that PMDD symptomatology reflects increased amygdala sensitivity to progesterone [56].

Limitations of this study included the relatively few participants and the lenient statistical threshold, warranting replication in a larger sample before the hypothesis of socially determined corticolimbic alterations in PMDD can be confirmed. Furthermore, only the contrast between social and non-social images with negative valence was analyzed, and future studies could disentangle the effect of each stimulus type by contrasting both types of images to more neutral slides. Strengths include the careful diagnostic procedure with prospective ratings of PMDD symptoms and direct estimates of progesterone as well as a methodology focusing on a theoretically defined brain territory with corresponding statistical small volume corrections for multiple comparisons.

## Conclusions

This pilot study indicates that aversive and socially relevant stimuli as compared to non-social aversive stimuli enhanced activity in affective processing brain regions that were functionally coupled to cognitive control areas with compromised activity. We therefore argue that patients with PMDD are characterized by altered corticolimbic circuit processing specifically in response to social emotions, and that progesterone in part influences corticolimbic processing by tuning emotion processing areas.

## Abbreviations

ACC: anterior cingulate cortex; BOLD: blood oxygenation level-dependent; fMRI: functional magnetic resonance imaging; IAPS: International Affective Pictures System; MADRS-S: Montgomery-Åsberg Depression Rating Scale -self rated; PMDD: premenstrual dysphoric disorder; PTSD: post-traumatic disorder; ROI: region of interest; STAI-S: State-Trait Anxiety Inventory -self-rated.

## Competing interests

ISP serves occasionally on advisory boards or acts as invited speaker at scientific meetings for MSD, Novo Nordisk, Bayer Health Care, and Lundbeck A/S. The other authors declare that they have no competing interests.

## Authors’ contributions

MG: design of study, data collection, statistical analyses, manuscript writing and final approval of the manuscript. VA: statistical analyses and final approval of the manuscript. EB: data collection and final approval of the manuscript. JW: data collection and final approval of the manuscript. ISP: design of study, financial support, data collection, critical revision and final approval of the manuscript. MF: design of study, financial support, statistical analyses, manuscript writing and final approval of the manuscript. All authors have read and approved the final manuscript.

## Supplementary Material

Additional file 1 **A schematic example of the used paradigm.** The paradigm included exposure to emotional images of negative or positive valence that were preceded by a cue indicating the upcoming valence. In our study, only BOLD reactivity while viewing images of negative valence with social and non-social content was studied. Click here for file

Additional file 2: Table S1 Valence and arousal ratings. A table including the ratings of pictorial stimuli on the IAPS nine-point visual analog scale for women with PMDD and healthy controls across the menstrual cycle. Click here for file
