# Supplementary figures and images for: Social stimulation and corticolimbic reactivity in premenstrual dysphoric disorder: a preliminary study
Source: Biol Mood Anxiety Disord. 2014 Feb 26;4:3. doi: 10.1186/2045-5380-4-3 (PMC4015856; doi:10.1186/2045-5380-4-3)

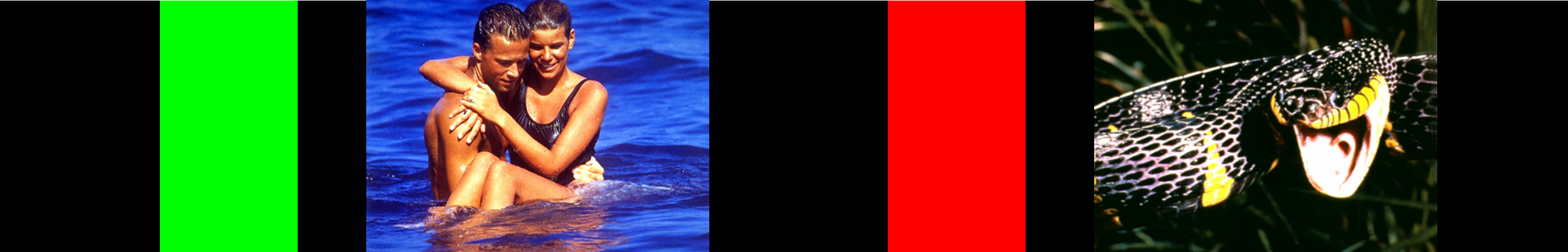

Supplement: Additional file 1 — A schematic example of the used paradigm. The paradigm included exposure to emotional images of negative or positive valence that were preceded by a cue indicating the upcoming valence. In our study, only BOLD reactivity while viewing images of negative valence with social and non-social content was studied. [file 2045-5380-4-3-S1.tiff]
