# Supplementary material for: Social stimulation and corticolimbic reactivity in premenstrual dysphoric disorder: a preliminary study
Source: Biol Mood Anxiety Disord. 2014 Feb 26;4:3. doi: 10.1186/2045-5380-4-3 (PMC4015856; doi:10.1186/2045-5380-4-3)
Supplement: Additional file 2: Table S1 — Valence and arousal ratings. A table including the ratings of pictorial stimuli on the IAPS nine-point visual analog scale for women with PMDD and healthy controls across the menstrual cycle. [file 2045-5380-4-3-S2.docx]

**Additional file 2 – Ratings of pictorial stimuli**

Ratings of pictorial stimuli on the IAPS 9 point visual analog scale [31] are given in supplementary table 1. Women with PMDD rated the social images as more negatively valenced than healthy controls, and more negative than non-social images in the luteal phase (see main text for statistics). Ratings of arousal were similar for social and non-social stimuli in both groups (both measures t (25) <0.45; p>0.17). There was no difference in arousal ratings between phases in any group (both measures t (13/14) <1.6; p>0.14).

**Table S1.**

Valence and arousal ratings (1-8) for the emotional pictures. For valence 1 indicates highly unpleasant and 8 highly pleasant. Higher arousal values indicates higher arousal.

|  | Healthy controls  (n = 13) | Women with PMDD  (n = 14) |
| --- | --- | --- |
| Social arousal follicular phase | 5.1 ± 1.9 | 6.1 ± 1.6 |
| Social arousal luteal phase | 5.3 ± 2.0 | 6.1 ± 1.7 |
| Non-social arousal follicular phase | 5.1 ± 1.8 | 5.7 ± 1.5 |
| Non-social arousal luteal phase | 4.9 ± 1.8 | 5.1 ± 0.7 |
| Social valence follicular phase | 2.5 ± 1.3 | 2.1 ± 0.7 |
| Social valence luteal phase | 3.1 ± 1.4 | 2.0 ± 1.7* |
| Non-social valence follicular phase | 3.1 ± 1.4 | 3.1 ± 1.1 |
| Non-social valence luteal phase | 3.1 ± 1.4 | 3.2 ± 0.7 |

*) rated as more negative than non-social stimuli and also relative social stimuli in controls
